# Influential Factors of Non-suicidal Self-Injury in an Eastern Cultural Context: A Qualitative Study From the Perspective of School Mental Health Professionals

**DOI:** 10.3389/fpsyt.2021.681985

**Published:** 2021-09-28

**Authors:** Xiaoyu Chen, Ying Zhou, Li Li, Yanfei Hou, Ding Liu, Xueling Yang, Xiaoyuan Zhang

**Affiliations:** ^1^Department of Psychology, School of Public Health, Southern Medical University, Guangzhou, China; ^2^Huangpu School, Shenzhen, China; ^3^Department of Humanities and Mental Nursing, School of Nursing, Southern Medical University, Guangzhou, China; ^4^College of Psychology, Shenzhen University, Shenzhen, China; ^5^Department of Psychiatry, Zhujiang Hospital, Southern Medical University, Guangzhou, China

**Keywords:** non-suicidal self-injury, adolescents, culture, school mental health professionals, qualitative study

## Abstract

**Background:** Adolescent non-suicidal self-injury (NSSI) is becoming a serious public health concern worldwide. In recent years, there has seen a significant increase in both the growth rate and cases of NSSI in Eastern countries, such as Japan, India, and China. In China, most schools have a mental health office that comprises mental health professionals (MHPs), who are the first to respond to student mental health problems, which include NSSI. MHPs possess comprehensive knowledge as well as unique insight into adolescent NSSI. However, very few studies on NSSI have incorporated their perspectives. In this work, we seek to add novel insight by conducting a study focusing on adolescent NSSI from the perspective of MHPs.

**Methods:** We recruited a total of 24 MHPs from different schools using purposive sampling and conducted a semi-structured interview on NSSI-related issues. Each interview was voice recorded and lasted ~30 min. A thematic analysis was performed for the responses to study the most common and concerning issues underlying NSSI.

**Results:** We extracted three major themes and eight sub-themes from the interview records, which included (1) the impact of Chinese culture on NSSI (sex-bias discrimination, overly high expectations, and inappropriate parenting style); (2) life events in school (internet use, academic pressure, and romantic relationships); and (3) opinions on the status quo of NSSI in China (ignorance and stigma).

**Conclusion:** Results showed that culturally sensitive influential factors, such as sex-bias discrimination, the imposing of unrealistic expectations for personal achievement, and inappropriate parenting style, should be given more attention to prevent, assess, and intervene in NSSI. Emerging factors of modern society and adolescent lifestyles, such as academic pressure, internet use, and romantic relationships, should also be considered for treatments.

## Introduction

Non-suicidal self-injury (NSSI) refers to the intentional damage of one's own body tissues without suicidal intention. This behavior is not socially sanctioned (1–3). It commonly takes the form of skin-cutting, burning, and severe scratching (4, 5). NSSI is particularly prevalent in adolescence (5) and begins to manifest between 12 and 14 years of age (6). In non-clinical samples, the global prevalence of NSSI among adolescents is ~17–18% (7, 8). However, several studies have reported that the estimated prevalence among middle school students is 22.37, 27.4, and 29% (9–11), which is considerably higher than the global NSSI rate among all populations.

Over the years, numerous studies have investigated NSSI behaviors and their causes. Most of these have approached the problem from two perspectives. The first is exploring the risk factors of NSSI and the degree to which they contribute to the possibility of engaging in NSSI (12). Several risk factors related to NSSI have been studied, which include individual factors, childhood experience, family functions, and social relationships. However, only previous history of NSSI, cluster B personality disorder, and hopelessness were shown to be the strongest risk factors. This highlights the current lack of robust risk factors for NSSI (10, 12). The primary issue is the fact that NSSI is related to the interactions between and the combined effect of numerous factors (13). Factors that have been identified to date remain poorly understood (12, 14), and results have been inconsistent (2, 15). Thus, further research is necessary to determine the risk factors and their combined effect on NSSI behaviors (2). In addition, most research on NSSI and associated factors have been focused on correlations based on surveys only (12, 16–18). This study aimed to offer new insight into such correlations and conduct an in-depth analysis of the underlying mechanism using qualitative methods.

Furthermore, most previous studies were performed in developed countries, and studies considering race, ethnicity, and culture in developing countries are scarce (19). For example, China, a developing Eastern country, has significantly different cultural and social value characteristics to those of developed Western societies; this will likely introduce a unique set of risk factors for NSSI. Previous research showed cultural factors might significantly impact the meanings, risk factors, and gender differences in NSSI (20), and research is required to investigate such culturally specific risks and protective factors of NSSI (21). Moreover, the mental health field in China began relatively late, in both clinical and educational settings. With 189 million school children and ~28 million undergraduate students in China, the mental health of Chinese adolescents who experience NSSI needs more attention (22), with a specific focus on factors correlated with NSSI in the context of Eastern society.

Few qualitative studies on NSSI have considered current mental health practices in schools. In China, schools are required to have a mental health office/department. The primary responsibilities of MHPs include providing mental health care classes, psychological counseling, prevention of mental illness, and crisis intervention for students. Generally, schools regularly perform mandatory mental health assessments for students and identify those at high risk of engaging in NSSI and other life-threatening behaviors. MHPs will be responsible for providing preventive intervention to these students. These can be psychotherapy or referral to other professional psychiatrists. In the event where it is found that a student engages in NSSI, MHPs are typically the first point of contact to call upon to handle the incident. For many students, they are the most crucial source of support. Therefore, they are in a unique position to identify and respond to NSSI behavior among youth, and they play an essential role in the education, prevention, and timely treatment of NSSI in schools (23, 24). Studying NSSI from school MHPs and understanding their experiences working with individuals and families in different schools and during various life events may be a valuable and crucial step toward gaining a comprehensive understanding of adolescent NSSI.

Meanwhile, the current studies on NSSI-associated factors have focused primarily on the family environment (25–27), childhood experiences (28–31), and mental health status (32–36). However, the school environment has not received much attention, despite being the location where most NSSIs occur. Incorporating the school context into the study is critical for understanding the issue (37). The lifestyles of adolescents in modern society undergo dramatic changes, and it is essential to determine whether these changes lead to changes in influential factors for NSSI. A qualitative approach is an appropriate method for exploring such issues.

Therefore, we conducted a culturally sensitive qualitative study of NSSI behavior in the context of the Chinese cultural background. We studied the influential factors of NSSI within a socio-cultural background from the perspective of school MHPs. Specifically, we aimed to identify several important contextual influencing factors related to NSSI, which might include gender, family, and culture. We also try to figure out and explained various events/factors in the school life of individuals that correlated and contributed to NSSI. Also, by exploring the attitudes, views, and understanding of NSSI of school MHPs, we could analyze the status quo of NSSI in China which could be used in the following-up prevention and interventions to help adolescents in the future.

## Methods

### Participants

We adopted the purposive sampling method. A total of 25 MHPs from school counseling associations who had previous experience in dealing with NSSI cases were recruited for an interview. Twenty-five participants came from the eastern province of China and two came from the countryside. One participant withdrew because of quarantining because of COVID-19. Thus, 24 MHPs completed the study. The study ran from February 2020 to June 2020 and was approved by the medical ethics committee of Southern Medical University. All participants were informed of the study's purpose, methods, and privacy and provided written informed consent. Details are provided in Table 1.

**Table 1:** Participant characteristics.

Age (SD, range)	35.5 (6.4, 23.0–49.0)
Years of working (SD, range)	10.2 (5.4, 2.0–18.0)
**Gender**	
Female	15 (62.5%)
Male	9 (37.5%)
**Education**	
Bachelor	9 (37.5%)
Master	11 (45.8%)
Doctor	4 (16.6%)
**Occupation**	
Primary school	4 (16.6%)
Middle school	16 (66.6%)
College	
**Region**	7 (29.1%)
City	22 (91.7%)
Countryside	2 (8.3%)

*SD, standard deviation*.

### Procedures

An in-depth and semi-structured interview was conducted in a private psychological therapy room. Participants were informed that following the interview, they will be asked demographic questions. The outline of our interview questions was designed according to existing literature and in-depth discussions with experienced school psychologists, psychiatrists, and clinical social workers working with adolescents engaging in NSSI (23, 26, 35). The interview covered seven main topics, such as: (1) What is your experience of dealing with NSSI cases? (2) Can you use three words to describe your overall impression of NSSI behavior? (3) What is your understanding and views regarding NSSI? (4) What do you think are the main factors that contribute to NSSI? (5) Are there any unique characteristics of NSSI in the context of Chinese culture? (6) Based on your experience, what do you think students engaging in NSSI have in common? During the interview, additional follow-up questions were asked to encourage elaborations on participants' statements. Each interview was voice-recorded and lasted ~30 min.

### Data Analysis

At the end of each interview, three researchers independently transcribed the voice recordings and encoded the data. The three researchers came from backgrounds of psychiatry, psychology, and clinical social work, respectively. General information was extracted into a table. Data coding was performed using NVivo software, version 12 Plus. For the thematic analysis, we followed the six-step process of Braun and Clarke (38). Subtheme analysis was performed by extracting the nodes repeatedly. For each subtheme, we calculated the percentage of participants who mentioned it. Intuitively, a higher rate suggested that the subtheme was more concerning. Results were carefully adjusted until all three researchers reached a consensus and reviewed by all staff in the department.

## Results

**Figure 1:**
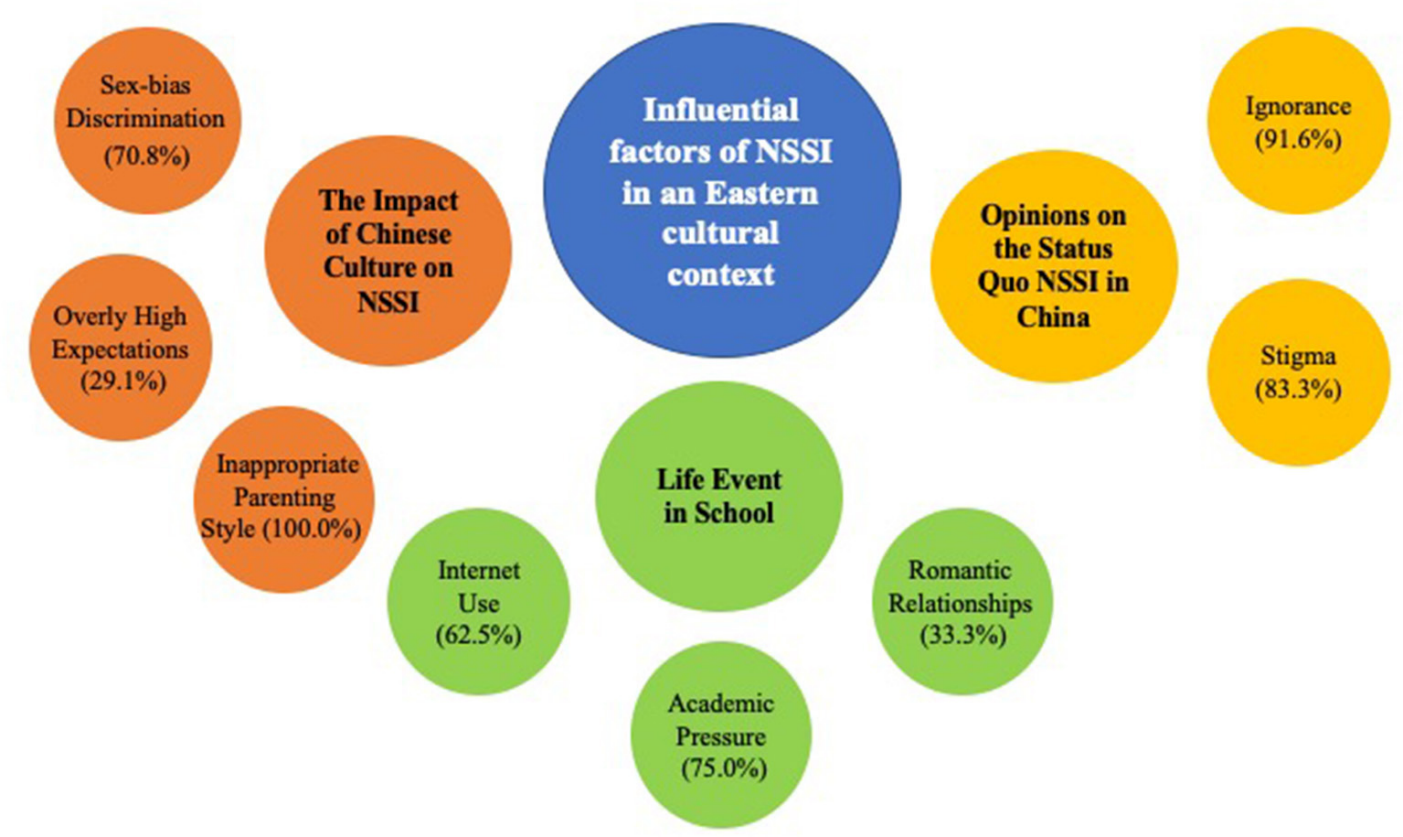
Thematic map of the three major themes (middle circle) and eight sub-themes (small circle).

Three major themes were extracted from the thematic analysis on the responses of the participants: (1) Impact of Chinese culture on NSSI, (2) life events in school, and (3) opinions on the status quo of NSSI in China (see Figure 1). Within each major theme, we also identified several sub-themes. The following is a summary of the thematic analysis results, which includes a sample of corresponding responses from participants.

### Theme 1: Impact of Chinese Culture on NSSI

#### Subtheme 1: Sex-Bias Discrimination (70.8%)

Sex-bias discrimination was a contributing factor to NSSI behavior, according to several participants. In some families, girls were long considered lower priority than boys and were subject to low self-esteem. Girls were under more pressure from parents' constant ignorance of their emotional needs, and for some, NSSI had become a coping strategy for dealing with their emotional and mental problems.

“*In some families, it is still possible that boys are held at higher importance over girls. As a result, girls may start suffering from low self-esteem from childhood. They tend to underestimate their own value a lot, which, in some, leads to NSSI…*” (p. 10).

“…*In some families with many children, parents may not treat each child with an equal amount of care. Some children who receive little care may engage in NSSI to gain more attention…*” (p. 11).

Some participants reported that this situation was more common in areas where a preference for sons was prevalent. It was usually girls at home who engaged in NSSI.

“…*the population here is mostly composed of families from Chaoshan, where the preference of sons over daughters is deeply rooted in their values. Some families have more than five children who all studied in the same school. Usually, children who engage in NSSI are girls. They receive little to no attention from their parents, yet, they are still responsible for undertaking family chores and various requests from the parents, including academic performance. In contrast, boys have very little pressure and worries and are offered an effortless life. As a result, girls are subject to much higher pressure and are more prone to NSSI…*” (p. 13).

“…*This kind of family generally has more children. Girls are not able to get sufficient attention…*” (p. 17).

“*Girls in families from Chaoshan generally have very low self-esteem. They are always ignored, which makes it easier for them to engage in NSSI behavior…*” (p. 21).

#### Subtheme 2: Overly High Expectations (29.1%)

Several participants mentioned that there is a deeply rooted value in Chinese culture that the next generation must be better than the parent generation. As a result, parents often have very high expectations of their children, especially in regard to academic achievement (39, 40). This can lead to enormous amounts of pressure and insufficient awareness of their children's mental health (41, 42) and may consist with NSSI behavior.

“…*When parents fail to achieve the life they want, they pass it onto their children, pushing them to work extremely hard, hoping that their children will be able to achieve what they were not able to…*” (p. 10).

“…*In Chinese culture, parents making children successful is highly valued, where each generation should be better and end up in a higher social class than their parents. Parents nowadays are increasingly anxious about this. To many parents, it is unacceptable that their children end up worse than them because their children were born in a much better time with much better material support…*” (p. 13).

“…*Parents pay exclusive attention to children's academic performance and tend to care less about their emotional and mental health needs…*” (p. 15).

#### Subtheme 3: Inappropriate Parenting Style (100.0%)

Many participants also reported that Chinese parents tend to be overly controlling, use an authoritative parenting style, and have little respect for their children. Some adolescents chose to harm themselves and engaged in NSSI after being subjected to such highly pressured parenting styles over a prolonged period as a way to rebel against their parents.

“…*In overly protective and controlling families, children do not dare stand up against their parents. When they are pressured and their feelings are suppressed for too long, they tend to resist through self-injurious behavior as a way to get back at their parents and make them feel guilty…*” (p. 4).

Participants also reported that many parents also held the idea of “spare the rod, spoil the child.” These parents believe that physical punishment is necessary to educate their children. Such practices caused severe psychological harm to adolescents. Moreover, this led children to punish themselves, which caused NSSI.

“…*In many NSSI cases, children had been abused by their parents previously. Even when they become teenagers, they may still be subject to physical punishment by their parents. Children exposed to such prolonged periods of abuse are more prone to engaging in NSSI…*” (p. 14).

In Chinese culture, tolerance and toughness are valued highly. Most participants stated that children were often encouraged and educated to suppress their feeling of sadness, fear, and anger. To some degree, this led to an increase in NSSI among children to seek relief from suppressed emotions.

“…*I once knew a child who was not allowed to cry in front of the family since childhood. Whenever he did so, his parents would lock him out until he stopped. This continued for more than a decade. The child always told himself not to cry. But sometimes, he was so depressed that he had no other way to cope with it. As a result, he started engaging in NSSI as a way to seek relief. Children were discouraged from expressing their feelings and emotions openly. They were usually taught to suppress them*.” (p. 2).

“*The lack of freedom in openly expressing emotions and feelings, the overly controlling and protective parenting style, and the practice of physical punishment in the family has resulted in children's use of extreme coping strategies for dealing with negative emotions and difficulties in their life, which in many cases led to NSSI*” (p. 24).

### Theme 2: Life Events in School

#### Subtheme 1: Internet Use (62.5%)

According to participants, the internet was a strong influence on children's NSSI behavior. Children can easily access a large amount of self-injury- and violence-related information on the internet. Some children thought it was simply “cool” and “fun” and imitated the NSSI behavior they saw on the internet.

“…*Because they can now get so much information on the internet, they just thought it (NSSI) was fun and cool and they gave it a try…*” (p. 11).

“…*When he saw this unusual behavior on the internet, he tried something similar out of curiosity…*” (p. 18).

“…*I feel there is some kind of herd mentality behind NSSI behavior, like chasing a trend. When students see reports and cases of NSSI from the internet or the news without proper guidance, they may develop a curiosity to follow the practice and try it out…*” (p. 12).

Some participants reported that there exist online NSSI communities where people share experiences, related topics, and methods of NSSI behavior. Children can easily and quickly join these communities, which exacerbates NSSI behavior.

“…*There are some communities on the internet. Teenagers interact with each other in this community and engage in self-injurious behaviors together…*” (p. 23).

“…*another point is that it is very common for two teenage users in these online communities to share similar experiences and thoughts regarding NSSI. This can be extremely dangerous as they tend to encourage each other further …*” (p. 9).

“…*the culture in these NSSI communities, especially for teenagers, can be very toxic. They are constantly exposed to simplistic, dark, and negative information. In addition, these communities tend to be very extreme and biased. Whenever someone posts negative views and emotions, others echo and agree. As a result, such negative information and ideas are reinforced among the participants. If anyone stands up or tries to correct and help make sense of the negative information, they will be ignored and dismissed. Such an extreme and dark environment can be very harmful to teenagers…*” (p. 4).

#### Subtheme 2: Academic Pressure (75.0%)

Most participants reported that overwhelming academic pressure, which is very common in elementary school, middle school, and college students, directly contributed to NSSI behaviors.

“…*It is common for teenagers to injure themselves to cope with academic pressure, regardless of their academic performance. In many cases, the pressure comes from parents being pushy and having high expectations…*” (p. 13).

“…*when teenagers engaged in NSSI, they were under great academic pressure most of the time…*” (p. 14).

“…*often grades are the trigger for NSSI. In many cases, they injure themselves when they did not do well on a test or when they were struggling with a subject…*” (p. 20).

#### Subtheme 3: Romantic Relationships (33.3%)

A few participants also mentioned that some teenagers engaged in self-harming behavior if they had romantic feelings for someone as an expression of love.

“…*For example, I once met a boy who had a crush on a girl. But for some reason, the girl hurt herself. The boy thought he could only be with her if they were the same. Then the boy also started to self-harm… Friends may also engage in self-injurious behavior together to demonstrate to others that they are in the same friend circle…*” (p. 13).

Some teenagers in relationships may use self-injurious behavior when they encounter conflicts.

“…*Sometimes when teenagers in a relationship fight, one may engage in self-harm to get attention and care from the other…*” (p. 20).

“…*Some teenagers may get their boyfriend's or girlfriend's attention by hurting themselves. When teenagers in a relationship argue, one of them would cut themselves to get the other's attention. Moreover, they want their boyfriend/girlfriend to care about them…*” (p. 20).

“…*Teenagers who are in a relationship may hurt themselves on purpose, take pictures, and post on social media as a threat to get his/her attention and care…*” (p. 13).

### Theme 3: Opinions on the Status Quo on NSSI in China

#### Subtheme 1: Ignorance (91.6%)

Many participants expressed that there was not sufficient awareness of NSSI behaviors in school.

“…*NSSI is given much less attention compared with suicide. This is likely because suicide causes death, which is a serious social event. The government usually has to impose censorship on the news when this occurs. However, for NSSI, many teachers consider it to be of little concern, and some even consider it simply as a student's trick for getting attention*” (p. 13).

In addition to the lack of awareness in school, some participants also reported that many parents lack basic mental health knowledge, which results in inappropriate perceptions of their children's NSSI behavior.

“…*Many parents consider their children to be fine as long as the problem is not severe enough to lead to suicide…*” (p. 22).

“…*Some parents simply don't consider NSSI as a problem at all…*” (p. 17)

“…*Her (a child who engaged in NSSI) teacher once reached out to her parents regarding her NSSI behavior. The parents were annoyed and did not show any concern. They even considered that their child was causing too much trouble for them. They also didn't believe that their child was depressed as they thought that whatever pressure their child was under, it was nothing compared with theirs…*” (p. 18).

#### Subtheme 2: Stigma (83.3%)

Many participants considered NSSI to be associated with social stigma. This hinders timely interventions for students who engage in NSSI. Many parents were reluctant to seek professional help and some denied that their children had any problems. Moreover, some parents were concerned that seeking professional help would expose their children to judgment from others, which may negatively impact them.

“…*Many parents feared that they will be stigmatized because of their children's psychological problems; a small number of parents tried to avoid talking about related problems…Some parents were not even willing to let their children receive psychological counseling…*” (p. 4).

“…*If you take him (child) to a psychiatrist, it puts a label on the child; a label that says he has a serious problem…*” (p. 3).

“…*One parent I talked to expressed that turning to an intervention, psychiatrists, or medical help of any kind is not the solution to NSSI. They thought that it is a problem of mindset and educating the child to have the correct mindset would address these problems. A lot of parents share this opinion. As a result, many parents don't support their children in seeking help from psychiatrists. They consider it to be the children's problem and that children should be responsible for themselves…*” (p. 17).

Fortunately, according to many participants, many students in later generations were not concerned about stigmatization as much as their parents were, and many of these students actively sought help from psychiatrists.

“*Students are aware of their psychological and personality problems, and they will actively seek help from teachers… some students will even proactively schedule time with teachers outside of school/working hours when they feel they need more counseling…*” (p. 11).

“…*Now, more and more students who have such problems are actively seeking help. Even when face-to-face counseling is not immediately available, they will still try to reach out to us about their problems using messages. Compared with previous decades, stigmatization seems to be getting better. Back in 2005 or 2006, many students feared being laughed at and judged if they sought help from psychiatrists…*” (p. 18).

“…*Some students feel relief when they are diagnosed. I once had a student who struggled with school and was quite depressed and confused. He started seeking help and was diagnosed with mild depression. To my surprise, the student was relieved after learning the diagnosis. To him, it was like he finally found someone who could identify his problem and help him get through it…*” (p. 18).

## Discussion

To the best of our knowledge, this is the first study to investigate adolescent NSSI behavior from the perspective of school MHPs in China. Our findings bridge the gap in the literature on the factors that influence Eastern socio-cultural backgrounds from the perspective of school MHPs. We found that sex-bias discrimination (70.8%), overly high expectations (29.1%), and inappropriate parenting style (100.0%) influenced adolescent NSSI behavior in China. In addition, teenagers who fall in love at an earlier age (33.3%), widespread internet use (62.5%), and overly high academic pressures (75.0%) also triggered NSSI behaviors.

Discussing NSSI in the cultural context is necessary. Although NSSI has been observed and described in almost all cultures, they do have different characteristics in different cultures. These include prevalence, gender difference, methods of self-injury, and motivation (19, 20). Given the close relationship between culture and NSSI behaviors, culture may significantly impact NSSI than previously assumed (19, 43, 44). According to the Diagnostic and Statistical Manual of Mental Disorders-5, culture is essential for defining, preventing, and intervening in NSSI behavior (2). However, the field of NSSI has been dominated by research conducted in Caucasian-majority samples in developed Western countries, such as the United States, Canada, Australia, and European countries, despite the higher estimated NSSI prevalence among Chinese adolescents than that worldwide (9, 19, 20). Clearly, there is an underrepresentation of non-Western cultures and ethnic/racial minority groups in the NSSI literature. The lack of inclusivity to some extent gives the wrong impression that NSSI shows itself in identical ways across countries, ethnicities, and cultures. Thus, research on the mental health of Chinese adolescents (22) who engage in NSSI behavior is critical and valuable, especially those that incorporate Eastern social values and cultural contexts in studying NSSI risk factors. This study aimed to fill this gap. We found sex-bias discrimination, overly high expectations, and inappropriate parenting styles may contributing to NSSI in China. However, further research involving the broader community is needed.

There has not been a consensus on the percentage of the population engaging in NSSI and whether and why there is any difference in NSSI patterns between genders (45–47). In the Chinese population, some research demonstrated no gender significant gender difference in NSSI behaviors (48–50), while some research found more girls than boys reported engaging in NSSI (10, 51). The sample type, age, and areas with different gender norms and cultures could partly influence the gender prevalence of NSSI (47). Existing quantitative studies have shown that the relative prevalence of NSSI among the male and female populations can vary in different settings. However, it remains an open question what the determining factors are. Through a qualitative study, we contribute to understanding this problem by showing how cultural backgrounds and social values can be one of these essential factors. In particular, we showed that the preference of boys over girls, a mindset that is still deeply rooted in many regions in China, has made NSSI much more severe and prevalent among girls compared to boys. In some areas of China, such as the Chaoshan area, the preference for sons over daughters is still deeply rooted in social value. In these regions, boys are given much more attention and care in life, whereas girls are often put under more pressure and carry more burdens and responsibilities than boys; moreover, they are more often ignored. Several participants in our study reported that NSSI becomes a way for these girls to cope with the pressure, gain attention, or get back at their parents. This suggests that sex-bias discrimination historically rooted in Eastern culture persists and is a non-trivial contributing factor of NSSI. Our findings are also consistent with previous reports of differences in risk factors and causes of self-harm between boys and girls (52). However, whether this is similar in Western societies and how sex-bias discrimination impacts NSSI in a broader context remains uncertain. Additional qualitative and quantitative studies on this issue are needed.

Notably, we found that both parents' and adolescents' excessive anxiety around academic performance influenced NSSI behavior. Previous research showed that excessive academic stress is related to psychological problems, such as anxiety and depression (41). Several other studies have also examined the association between NSSI behavior in Western college students and academic performance (53–55). In the Chinese adolescent population, who have a higher academic burden, academic performance could be a crucial trigger for NSSI. Parents hold very high expectations for their children and force their children to study extremely hard, to the degree where academic performance is considered significantly more important than psychological well-being. Therefore, parents and school personnel must put the academic performance into perspective; sacrificing children's mental health or causing self-injurious behavior in children in exchange for high academic achievement is not recommended.

Previous studies have shown a positive correlation between excessive internet use and NSSI. The results of our study can explain this finding. With widespread internet use, teenagers are increasingly searching for, discussing, and publicly showcasing their NSSI experience on social media. Studies have shown that the internet both catalyzes and reduces NSSI behavior (56–58). According to school MHPs, the internet increases adolescents' access to NSSI and uncensored violence-related material, which can arouse their curiosity. Some teenagers view NSSI behaviors as “cool” and “fun” and imitate the NSSI behaviors they viewed on the internet. In addition, many join NSSI communities to share experiences, related topics, and methods of NSSI behavior. Children can easily join these communities, which likely exacerbates NSSI behavior. However, the internet can also be used to intervene and prevent NSSI behavior in teenagers. Research can be conducted to determine the type of content that should be blocked or censored for individuals who self-harm and information that could be offered to teenagers who are interested in searching for self-harm content online. Mental health services should consider assessing, understanding, and intervening in the online behavior of adolescents who engage in NSSI behavior. Although guidelines for MHPs have been suggested (34, 35), more extensive qualitative and quantitative studies are required to gain a full understanding of these influential factors.

Our result showed that NSSI receives insufficient attention in both school and family settings. This is primarily because NSSI is not a life-threatening behavior compared with suicidal behavior, which has led school staff and parents to not take NSSI as seriously. In addition to physical harm, the lack of attention and awareness is threatening the overall mental well-being of adolescents. Furthermore, the problem was compounded by the stigmatization (83.3%) of mental health disorders in China, which often results in parents ignoring and refusing mental health treatment for their children who engage in NSSI behavior. Such misunderstandings and perceptions of NSSI and other mental health disorders are consistent with the findings of previous research (26, 59, 60). There is an urgent need for a higher level of attention and psychoeducation that is accessible across various channels for both parents and staff.

A positive finding from our study is that according to MHPs, the later generation of adolescents perceives NSSI and mental disorders differently from their parents. They tend to possess more open and positive attitudes toward NSSI and mental disorders, which positively influences their awareness of their problems and promotes help-seeking behavior. The exact mechanism underlying how different perceptions of NSSI and mental disorders determine help-seeking behavior, and whether there is a causal relationship, remains uncertain. Exploring adolescent perspectives may offer significant benefits to the prevention and treatment of mental disorders, and this may be an avenue of future research.

## Conclusion

This study explored the influential factors of NSSI in the Eastern cultural context from the perspective of school MHPs. Our results showed that sex-bias discrimination, overly high expectations from the parent, high-pressure parenting style are the most significant factors contributing to NSSI among Chinese adolescents. The preference of boys over girls might make NSSI much more severe and prevalent among some girls compared to boys. Most parents in China have very high expectations for their children, especially in academic achievement. Over-controlling and authoritative parenting are common parenting styles in many of the families involved in our study. Children's mental health needs urgent attention from parents. In addition, our study suggests that researchers and clinicians should pay attention to how internet use, academic pressure, and romantic relationships can be related to NSSI. In particular, academic pressure is a trending cause of anxiety nowadays. Involution in adolescents' education has been a heated topic in recent years. Therefore, in future research, more quantitative research can be conducted to investigate the mechanism of how these factors contribute to NSSI and how they should be addressed in prevention and intervention. Besides, it is also essential to emphasize necessary psychoeducation for parents to address the negligence of mental health care needs for children.

## Data Availability Statement

The raw data is related to the subjects' personal privacy. Requests to access these datasets should be directed to Xiaoyu Chen, xiaoyupsychology@163.com.

## Ethics Statement

The studies involving human participants were reviewed and approved by the Medical Ethics committee of the Southern Medical University. The patients/participants provided their written informed consent to participate in this study.

## Author Contributions

XC and DL designed the study and wrote the protocol. LL, YZ, and YH assisted with the survey and managed the literature searches and analyses. XC, YZ, YH, and XY performed the data analysis. XC wrote the first draft of the manuscript. XY and XZ edited the final manuscript. All authors contributed, read, and approved the final manuscript.

## Funding

This work was supported by the National Natural Science Foundation of China (grant number: 72074105), the National Natural Science Foundation of China (grant number: 31800928), and the Natural Science Foundation of Guangdong, China (grant number: 2018A0303130044).

## Conflict of Interest

The authors declare that the research was conducted in the absence of any commercial or financial relationships that could be construed as a potential conflict of interest.

## Publisher's Note

All claims expressed in this article are solely those of the authors and do not necessarily represent those of their affiliated organizations, or those of the publisher, the editors and the reviewers. Any product that may be evaluated in this article, or claim that may be made by its manufacturer, is not guaranteed or endorsed by the publisher.
